# Balance bridges upstream deficits associated with falls in older adults: evidence from the National Health and Aging Trends Study Round 13 cohort

**DOI:** 10.1093/geroni/igag076

**Published:** 2026-07-01

**Authors:** Somayeh Shahsavarani, Yihsin Tai

**Affiliations:** Department of Audiology, San Jose State University, San Jose, California, United States; Department of Speech Pathology and Audiology, Ball State University, Muncie, Indiana, United States

**Keywords:** Postural control, Mental health, Hearing, Cognition, Hypertension

## Abstract

**Background and Objectives:**

Falls in older adults are a major public health concern, but the mechanistic pathways linking sensory, cognitive, and vascular deficits to falls are poorly understood. We hypothesized that balance acts as a critical mediator, channeling these upstream deficits into functional fall risk.

**Methods:**

Using cross-sectional data from 4805 community-dwelling adults (≥ 65 years) in the National Health and Aging Trends Study (NHATS) Round 13, we examined predictors of a composite fall-risk score (self-reported past falls and worry about falling). Survey-weighted multiple regression identified direct predictors, while mediation analyses tested the indirect effects of hearing, cognition, and vascular conditions through subjective (self-reported problems) and objective (performance-based) balance measures.

**Results:**

Subjective balance was the strongest direct predictor of fall risk (*β *= 0.53, *p *< .001), with additional direct effects from objective balance, depression, anxiety, and heart disease. Notably, hearing ability, cognitive function, and hypertension were not direct predictors of fall risk; their influence was fully or partially mediated by balance, establishing postural control as the primary pathway through which these upstream factors contribute to falls.

**Discussion and Implications:**

These findings identify a dual-pathway fall risk model driven by subjective and objective balance. While psychological factors and heart disease directly influence fall risk, upstream sensory, cognitive, and hypertensive deficits operate through balance as a convergent mediating pathway. Clinical fall prevention must prioritize subjective and objective balance measures, while managing mental health as a direct threat and addressing sensory and cognitive function as foundational to postural control.

Innovation and Translational Significance: Current fall prevention often addresses balance deficits in isolation. This study proposes a dual-pathway identifying self-reported balance problems as the primary driver of fall risk, with objective balance performance, mental health, and heart disease acting as additional direct predictors. Balance function also acts as a bridge for the indirect effects of hearing loss, cognitive decline, and hypertension. These results support a holistic approach to care: screening for balance (both self-perceived and objective), mental health, hearing, cognitive, and cardiovascular deficits as direct and upstream contributors to falls. This framework offers a pathway to refine fall prediction and enhance independence in aging.

## Introduction

Falls are a leading cause of premature morbidity and mortality among older adults, often resulting in severe injuries, diminished independence, reduced quality of life, and substantial financial burdens.^1^^,^^2^ As the global population ages rapidly, falls among older adults represent an escalating public health concern. Data from the National Center for Health Statistics suggest that in 2023, 41400 deaths of U.S. adults aged 65 and older were caused by unintentional falls.^3^ With the U.S. being one of the countries with high fall prevalence,^4^ the economic impact is undoubtedly profound: in 2020 alone, non-fatal falls generated healthcare costs of approximately US $80 billion, with the majority of these expenses covered by Medicare.^2^ Given the ongoing demographic shifts, the incidence and financial burden of falls are projected to increase, underscoring the urgent need for effective, evidence-based prevention strategies that target modifiable fall risk factors.

While balance dysfunction is a primary driver of falls in older adults, it is not an isolated deficit.^5^ Maintaining postural stability is a complex process requiring integrated inputs from multiple physiological domains such as hearing and vestibular systems, cognition, and psychological well-being.^5–7^ Aging induces widespread changes across these domains, impairing the integration of sensory information and the higher-level brain functions essential for postural control.^8^ Underpinning the integrity of this entire network is the health of the vascular system, a critical but often overlooked component. Age-related vascular changes, such as increased arterial stiffness and hypertension, can compromise neurovascular integrity, subtly undermining the sensory and neural systems responsible for postural stability.^9^ A comprehensive perspective suggests that deficits in sensory, cognitive, and vascular systems cause a decline in immediate balance function.^10^ The high co-occurrence of vestibular and cognitive impairment in older adults who fall, for instance, points to a shared vulnerability, not coincidental deficits.^11^ Therefore, a comprehensive model for fall prediction must consider how balance acts as a clinical nexus, bridging sensory and cognitive functions and their foundational vascular health.

Understanding the multifaceted nature of fall risk requires examining the contribution of each physiological domain. Hearing, for example, has been associated with gait control and fall risk.^12^ This association likely operates through several key mechanisms. One is the anatomical connection between the auditory and vestibular systems, where a common cause, such as aging, may result in concomitant dysfunction.^13^ Another mechanism involves perceiving environmental sounds to create a spatial map, or an “auditory anchor” that reduces postural sway.^14^ One other mechanism centers on cognitive load: with impaired hearing, greater cognitive resources are used to process auditory signals and maintain environmental awareness, which diverts finite attentional resources from postural control, thereby increasing gait variability and fall risk.^12–14^

Cognitive processes are also essential for safe ambulation and fall prevention. Executive functions, such as processing speed, attention, and cognitive flexibility, are critical for cognitive-motor integration, which allows planning and executing movements based on environmental demands.^15^^,^^16^ Executive function deficits, manifest as reduced ability to inhibit inappropriate motor responses, switch between tasks, and process visual-spatial information, are consistently associated with increased fall risk.^17^^,^^18^ The contribution of memory to postural control, on the contrary, is not well-established, especially when memory is assessed as a global construct.^18^^,^^19^ A more granular analysis reveals specific memory domains being critical predictors of falls. For example, immediate memory for real-time environmental encoding is necessary to guide safe movement.^20^ Furthermore, delayed memory performance has been shown to distinguish fallers from nonfallers,^19^ which suggests that recalling past spatial or motor experiences is a key protective factor. In addition, cognitive orientation generally represents a foundational awareness of time and current events, which serves as an essential indicator of global mental status.^18^ Deficits in cognitive orientation often indicate broader cognitive decline that impairs the higher-level sensorimotor and brain functions and disrupts the timely multisensory integration required for postural control.^5^^,^^18^ Therefore, a comprehensive model of fall risk must account for executive functions, the distinct contributions of specific memory domains, and cognitive orientation.

Mental health problems such as depression and anxiety are prevalent among older adults^21^ and are known independent risk factors for falls, even after accounting for physical health status.^22^^,^^23^ The mechanisms underlying this relationship are multifaceted. Psychological distress can impair cognitive shifting from an ongoing task to balance-related processes that are crucial for responding to postural perturbations.^24^ Furthermore, depression can reduce motivation for physical activity that helps maintain strength and balance and is often associated with psychotropic medications that may cause dizziness, further elevating fall risk.^23^ Beyond cognitive impacts, psychological distress manifests in maladaptive physical adaptations that compromise postural stability.^23^^,^^25^ Older adults experiencing anxiety or a fear of falling often adopt a reflexive “stiffening strategy,” characterized by lower-limb muscle co-contraction and reduced range of motion.^23^^,^^25^ While intended to stabilize posture, this behavior paradoxically increases instability by reducing the body’s ability to respond fluidly to external perturbations, thereby increasing gait variability.^25^^,^^26^ Furthermore, persistent distress frequently precipitates “activity avoidance,” where an enduring preoccupation with falling leads to the restriction of social and physical activities.^27^ This initiates a vicious cycle: restricted activity leads to a loss of muscle strength, which further degrades balance systems and reinforces fear, substantially elevating fall risk.^27^^,^^28^

Finally, vascular health impacts the neurological systems governing balance via at least 2 distinct pathways. One is acute blood pressure regulation. Maintaining hemodynamic stability during postural changes relies on the vestibulo-sympathetic reflex (VSR), a mechanism in which the vestibular system plays an integral role.^29^ Age-related vestibular decline can disrupt VSR, causing orthostatic hypotension (a sudden blood pressure drop upon standing), which is a significant fall risk factor.^30^ Beyond acute regulation, chronic vascular conditions, such as hypertension, pose a more insidious risk.^31^^,^^32^ However, in older adults, the high prevalence of confounding factors such as sensory deficit, cognitive decline, and polypharmacy often makes it challenging to identify the independent contribution of cardiovascular disorders to falls.^32^

Although these risk factors are well-documented, most research treats them as independent contributors,^8^ failing to capture the interconnected architecture of fall risk. A systems-level analysis, therefore, is necessary to deconstruct these relationships and identify upstream drivers. The National Health and Aging Trends Study (NHATS) provides a unique dataset of a large cohort^33^ to establish an integrated fall model. Following the National Institute on Aging workshop priorities on aging and balance,^34^ the predictors were categorized into 4 critical contributors to postural control: sensory (hearing), central neural (cognition), psychological (mental health), and cardiovascular (vascular conditions). While musculoskeletal and mobility-related factors are fundamental to the motor execution of balance,^34^ they were not modeled as primary predictors in the current study. Instead, self-reported arthritis and osteoporosis were included as covariates to partially account for their established structural and mechanical constraints on mobility. Similarly, while vision serves as fundamental sensory input for balance, it was used as a covariate to isolate the independent contributions of hearing.

This study aimed to disentangle the direct and indirect contributions of hearing, cognitive, mental health, and cardiovascular conditions to fall risk. We hypothesized that these domains affect fall risk through distinct pathways: mental health is directly associated with fall risk by disrupting attentional resources required for immediate postural control,^24^ whereas hearing loss and cognitive decline are indirectly associated with fall risk by impairing the higher-level processes governing motor control,^10^^,^^13^ operating as upstream factors that degrade postural stability. Furthermore, based on their established role in acute hemodynamic instability, we hypothesized that cardiovascular conditions act as direct predictors of increased fall risk. Through testing this mediating role, we aimed to clarify the indirect pathways, particularly for early-stage cognitive and cardiovascular contributors, to inform targeted, multimodal fall prevention strategies before the onset of any condition that independently increases fall risk.

## Methods

### Study design and population

This study used a cross-sectional design employing the NHATS Round 13 (year 2023) data. The NHATS is a prospective, longitudinal study of a nationally representative sample of Medicare beneficiaries aged 65 and older in the U.S. Begun in 2011 with annual data collection, NHATS is designed to foster research to reduce disability, maximize health and independent functioning, and enhance older adults’ quality of life. NHATS is sponsored by the National Institute on Aging (grant number NIA U01AG032947) and has been conducted by Johns Hopkins University, with the protocol approved by the Johns Hopkins University Institutional Review Board (protocol number 2083) and data collected by the research firm Westat. As the data analysis used publicly available, de-identified data, it was exempt from further institutional review.

**Figure 1:**
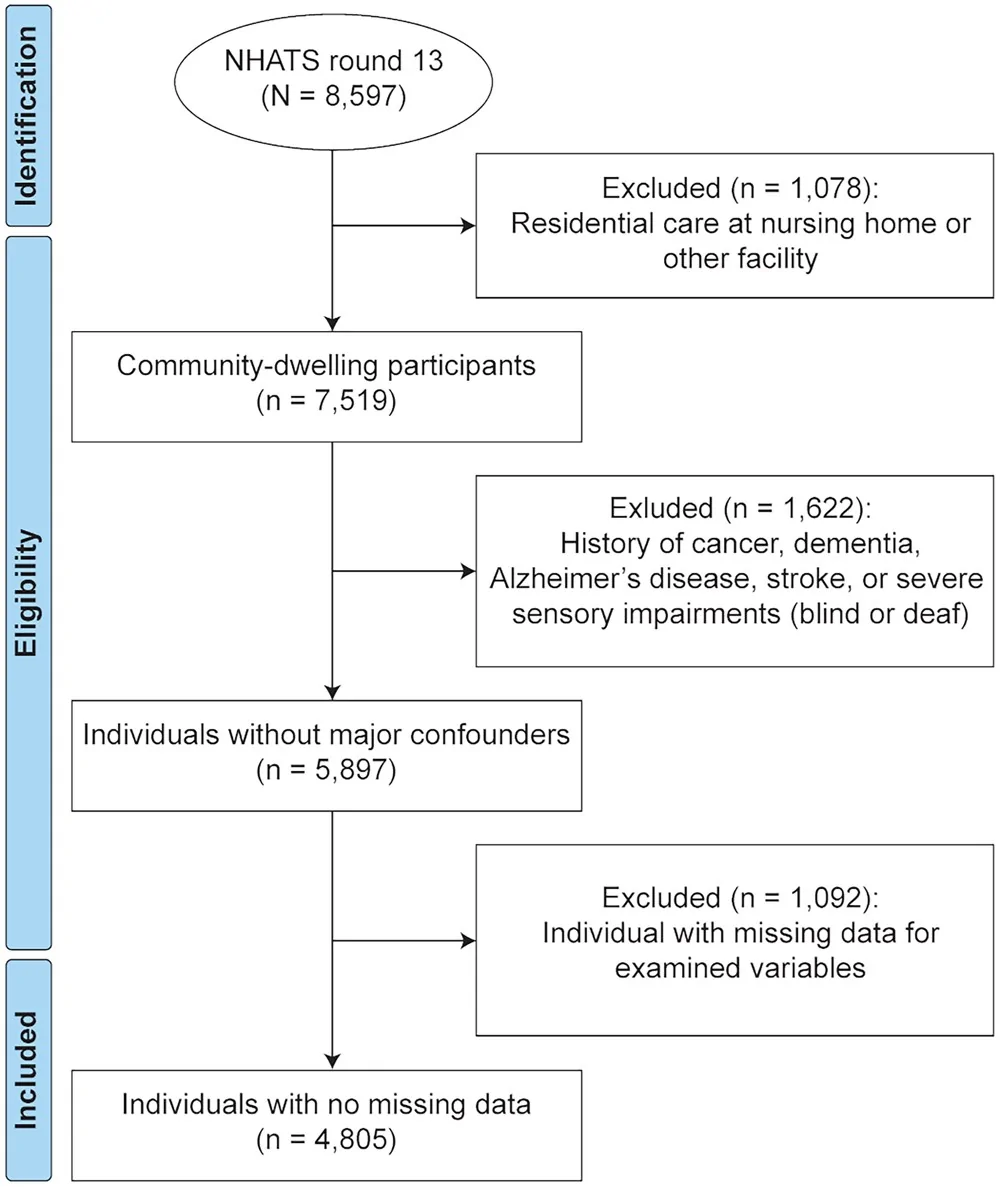
Flow diagram of sample selection from the National Health and Aging Trends Study (NHATS), Round 13 data. The figure details the exclusion criteria and the resulting sample sizes at each stage, leading to the final analytical sample of 4805 individuals.

The NHATS Round 13 Sample Person interview data include a replenished cohort in addition to the previous cohorts from 2011 and 2015, totaling 8597 samples and 1517 variables in the dataset. The present analysis was limited to community-dwelling individuals, resulting in 7519 respondents. From this sample, individuals with a self-reported history of cancer, dementia, Alzheimer’s disease, or stroke were excluded. Although these conditions are independently associated with increased fall risk, including them would have obscured the more subtle cognitive and vascular contributors in the general aging population. This exclusion allows the model to more accurately capture the insidious declines that precede major clinical diagnoses. Participants who reported being blind or deaf were also excluded to avoid confounding from severe sensory impairments. After applying these exclusion criteria and performing listwise deletion of missing data, the final analytic sample included 4805 community-dwelling older adults (Figure 1).

To assess potential selection bias, we compared the demographic and health profiles of the final analytic sample with those excluded due to missing data (*n *= 1092). Statistical comparisons (Supplementary Table 1) showed the excluded group was slightly older, with more females, lower education levels, and a higher prevalence of chronic conditions. Although indicating a selection bias, absolute differences were modest, with the median absolute difference of 3.7% (range: 0.1-9.6).

### Variables

All selected variables were extracted from the NHATS Round 13 dataset. For categorical variables, non-valid responses of “Missing (-9),” “Don’t Know (-8),” “Refused (-7),” and “Inapplicable (−1)” across all variables were systematically recoded as missing values (NaN) to facilitate listwise deletion. For composite measures, a final value was computed only if valid data were present for every underlying component item; otherwise, the construct was treated as missing.

#### Outcome variable: fall risk

The primary outcome was a fall-risk score. In NHATS, falling down is defined as “any fall, slip, or trip in which you lose your balance and land on the floor or ground or at a lower level.” Fall risk was operationalized using a composite score from 3 self-reported dichotomous variables (see Supplementary Methods for the conceptual rationale): falling in the last month, worrying about falling in the last month, and falling in the last 12 months. These variables were re-coded to 1 (Yes) and 0 (No) and summed for each participant, yielding a final fall-risk score ranging from 0 to 3, where higher values indicate greater fall risk. A score was computed only for participants with complete data on all 3 items. To ensure the primary predictor (self-reported balance problems) and this outcome variable (including “worry about falling”) did not overlap significantly, a weighted correlation analysis was conducted. The analysis revealed a moderate correlation (*r *= 0.45, *p *< .001), indicating the variables share only approximately 20% variance, confirming they are statistically distinct.

#### Predictor variables

##### Hearing

Hearing status was characterized by a summed hearing ability score based on 2 self-reported items: hearing on a telephone and carrying on a conversation in background noise. Each item was converted from the 1 (Yes)/2 (No) format to an analytical coding of 1 (Yes)/0 (No). This process yielded a summed score from 0 to 2, where a higher score indicates better functional hearing ability.

##### Cognitive function

Cognitive function was evaluated across 3 distinct domains: executive function, memory, and orientation. Executive function was measured by the Clock Drawing Test (CDT), with a score ranging from 0 (a clock that is not recognizable, indicating severe impairment) to 5 (accurate depiction of a clock, indicating no impairment). Memory was assessed through tests of immediate and delayed word recall. Participants repeated a list of 10 words immediately and recalled the words after completing other cognitive function tasks, with 10 as the maximum possible score for each task. Orientation was determined using a composite of 8 dichotomous items assessing knowledge of the current date and names of the current president/vice president (maximum score = 8). Executive function, immediate and delayed memory, and orientation scores were included as individual predictors.

##### Mental health

Mental health was assessed using the 4-item Patient Health Questionnaire-4 (PHQ-4).^35^ Each item was rated on a 4-point scale (1 = not at all; 4 = nearly every day), with higher values indicating greater symptom severity. From these ratings, 2 separate scores (range 2-8) were created: a depression score (sum of 2 depressive symptom items) and an anxiety score (sum of 2 anxiety symptom items).

##### Selected cardiovascular conditions

Cardiovascular condition was represented by 3 separate, self-reported dichotomous variables indicating a history of heart attack, heart disease, or hypertension. For each condition, any response of “Previously reported” was recoded to “Yes” to capture a lifetime history.

##### Balance

Balance was assessed using 2 complementary measures: a subjective self-report and an objective, performance-based test. The subjective measure captured whether a participant reported having balance/coordination problems in the past month (1 = Yes, 2 = No, recoded to 1 = Yes, 0 = No for analysis). The objective measure was the score from the balance subset of the NHATS expanded short physical performance battery (SPPB). The NHATS balance subset assesses balance in 4 standing positions: side-by-side stand, semi-tandem stand, full-tandem stand, and one-leg eyes open stand. The score has integer values between 0 and 4, with a higher score representing a better performance of the activities.^36^

The association between self-reported balance problems and the score of the NHATS SPPB balance score was assessed using a survey-weighted correlation analysis (see Supplementary Methods). A moderate inverse relationship (*r *=* −*0.33, *p *< .001) was found between self-reported balance problems and the objective score (see Supplementary Figure 1 for a boxplot illustrating the distribution of scores). The coefficient of determination (*r*^2^ ≈ 0.11) indicates that only about 11% of objective score variability is “explained” by self-report, suggesting the 2 measures capture related but distinct facets of balance function. As a result, both self-reported and objective balance measures were retained in all subsequent analyses.

#### Covariates

The analysis adjusted for several demographic, health, and functioning variables to control for potential confounders. Demographic covariates included age (6 ranges from 65 to 90+ years), gender (male/female), and highest education level completed (9 categories from no schooling to Master’s or above). Race was not included as a covariate because prior NHATS research^37^ shows it is significantly associated with both fall risk and SPPB performance; including it could lead to over-adjustment and obscure the isolated contributions of the primary variables. Health-related covariates included other chronic diseases (arthritis, osteoporosis, and lung disease), with “Previously reported” answers recoded to “Yes.” Additionally, functional vision was assessed with a visual ability score. This score was the sum of 2 dichotomous (Yes/No) self-report questions assessing the ability to read newspaper print and to recognize someone across the street, where a higher score indicates better functional vision. Functional vision was used as a covariate. Given that vision is a primary sensory input for balance, this ensures that the observed associations for other domains are independent of baseline visual deficits.

### Statistical analysis

All statistical analyses were conducted using Python (version 3.12.11; Python Software Foundation) in the Spyder Integrated Development Environment. The NHATS final analytic weights for Round 13 (variable name = w13anfinwgt0) were applied to all analyses to account for the complex survey design and to ensure estimates were representative of the U.S. older adult population. A *p*-value of <.05 was considered statistically significant.

#### Multiple regression analysis

A multiple linear regression was first conducted to examine the direct associations between the predictors and the fall-risk score. The predictors included hearing ability, cognitive function (executive function, immediate and delayed memory, and orientation), balance (self-reported and objective), mental health (depression and anxiety symptoms), and vascular conditions (heart attack, heart disease, and hypertension). The analysis used survey-weighted least squares (WLS) regression with heteroskedasticity-consistent 3 (HC3) robust standard errors, adjusted for all covariates (age, gender, education, vision, arthritis, osteoporosis, and lung disease). Survey-weighted linear regression was selected as the primary analytical approach to provide clinically interpretable estimates, with robustness confirmed via ordinal sensitivity analysis (see Supplementary Methods). Before fitting the model, multicollinearity was assessed by computing variance inflation factors (VIFs) for all predictors and covariates. All VIFs were well below the common concern threshold of 5 (see Supplementary Table 2 for all VIFs), suggesting that multicollinearity was not a significant issue in the model.

#### Mediation analysis

Predictors not significantly associated with fall risk in the full model were examined for mediation by balance (subjective and objective) using parallel mediation models. These analyses were conducted using survey-WLS regression with HC3 robust standard errors. The indirect effect (Predictor **→** Balance **→** Fall Risk) was evaluated using the Sobel test to accommodate the complex survey-weighted design (see Supplementary Methods). Mediation was classified as full or partial based on the significance of the direct and indirect pathways.

## Results

### Sample characteristics

A summary of participant demographics is provided in Table 1, and descriptive statistics for predictor means are detailed in Table 2. The sample was predominantly female (56.09%), with the largest age group being 70-74 years (34.94%), and a high school diploma being the most common education level (21.27%). The cohort’s mean survey-weighted fall-risk score was 0.50 (SD = 0.67).

**Table 1:** Sample characteristics, the NHATS Round 13 data (*n *= 4805).

Characteristics	Unweighted *n*	Weighted %^a^
**Gender**		
Male	2054	43.91
Female	2751	56.09
**Age (years)**		
65-69	650	21.97
70-74	1172	34.94
75-79	1207	22.22
80-84	973	13.23
85-89	506	5.30
≥90	297	2.34
**Education**		
No schooling completed	46	0.37
1st-8th grade	364	3.91
9th-12th grade (no diploma)	406	5.99
High school graduate	1091	21.27
Vocational diploma	356	7.17
Some college but no degree	711	15.83
Associate’s degree	340	8.19
Bachelor’s degree	750	18.66
Master’s or above	741	18.61
**History of chronic conditions**		
Arthritis (yes)	2974	56.47
Osteoporosis (yes)	1304	24.59
Lung disease (yes)	894	17.49
Heart attack (yes)	175	4.00
Heart disease (yes)	864	15.47
Hypertension (yes)	3309	62.66
**Balance**		
Balance or coordination problems (yes)	1390	24.72

Abbreviation: NHATS, National Health and Aging Trends Study.

a Percentages given among the nonmissing data for all variables unless otherwise noted.

**Table 2:** Unweighted and survey-weighted descriptive statistics for the fall-risk score and predictor scores, the NHATS Round 13 data (*n *= 4805).

Variables	Max score	Unweighted mean (*SD*)	Weighted mean (*SD*)
Fall risk	3	0.53 (0.69)	0.50 (0.67)
Hearing ability	2	1.89 (0.34)	1.91 (0.30)
Executive function	5	3.94 (1.06)	4.09 (1.01)
Immediate memory	10	4.97 (1.64)	5.31 (1.59)
Delayed memory	9	3.57 (2.00)	3.95 (1.94)
Orientation	8	6.85 (1.55)	7.11 (1.33)
Objective balance	4	2.40 (1.19)	2.65 (1.14)
Depression	8	2.81 (1.26)	2.77 (1.24)
Anxiety	8	2.73 (1.18)	2.70 (1.13)

Abbreviation: NHATS, National Health and Aging Trends Study.

### Direct predictors of fall risk

**Figure 2:**
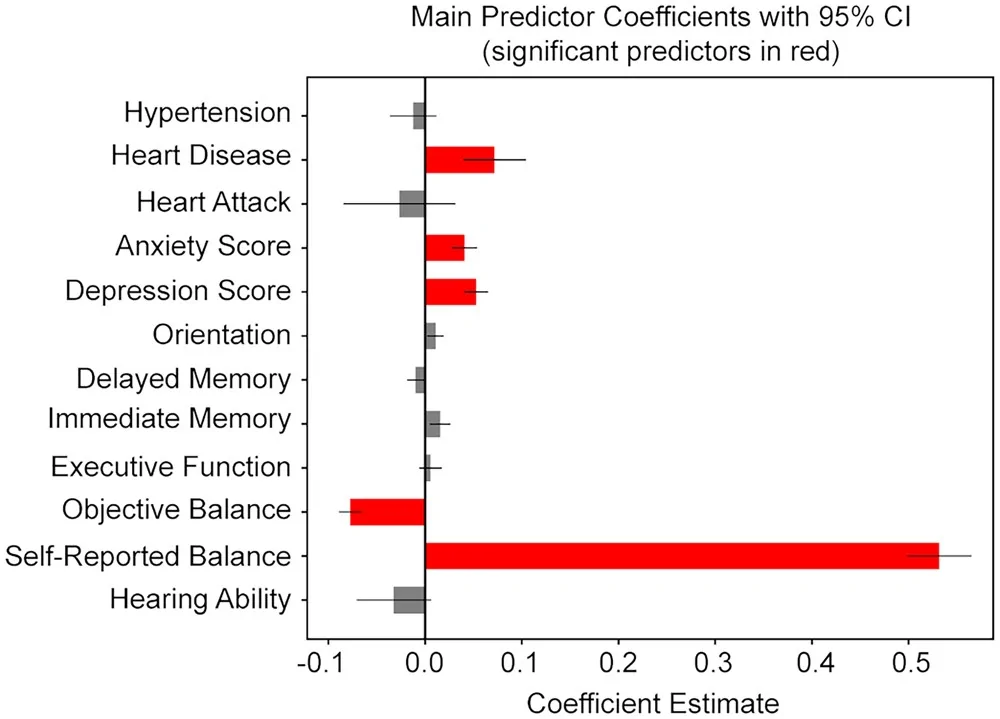
Coefficient estimates from the survey-weighted multiple linear regression model predicting the fall-risk score. Bars represent the coefficient estimate (*β*), and error bars indicate the 95% confidence interval. Red bars denote statistically significant predictors (*p *< .05).

The multiple regression model was statistically significant, explaining 26.5% of the fall-risk score variance (adjusted *R*^2^ = 0.26; *F*(19, 4785) = 52.06, *p *< .001). As shown in Figure 2, a self-reported balance problem had the largest effect on increasing the fall-risk score (*β*  =  0.53, *p *< .001). Additionally, better objective performance on the NHATS SPPB balance subset was significantly associated with a lower fall risk score (*β* = −0.08, *p *< .001). Both depression (*β*  =  0.05, *p *< .001) and anxiety (*β*  =  0.04, *p *< .01) symptoms were independently associated with a greater fall-risk score. Among vascular conditions, heart disease remained a modest but significant risk factor (*β*  =  0.07, *p *= .03). After accounting for all other factors, hearing ability, all cognitive scores, heart attack, and hypertension were not significant direct predictors of fall risk.

The conceptual rationale for the composite fall-risk score, alongside sensitivity analyses evaluating its individual components, are detailed in the Supplementary Methods. These analyses yielded consistent directional associations, confirming the analytical robustness of the composite measure (Supplementary Table 3).

### The mediating role of balance

Mediation analyses were conducted to test whether the non-significant predictors (hearing ability, cognition, hypertension, and heart attack) influenced fall risk indirectly through balance. Separate mediation models were conducted for each cognitive domain. Results revealed balance as a powerful mediator (see Supplementary Figures 2 and 3 for the magnitude and significance of the indirect effects mediated by self-reported and objective balance measures).

#### Mediation via self-reported balance problem

**Figure 3:**
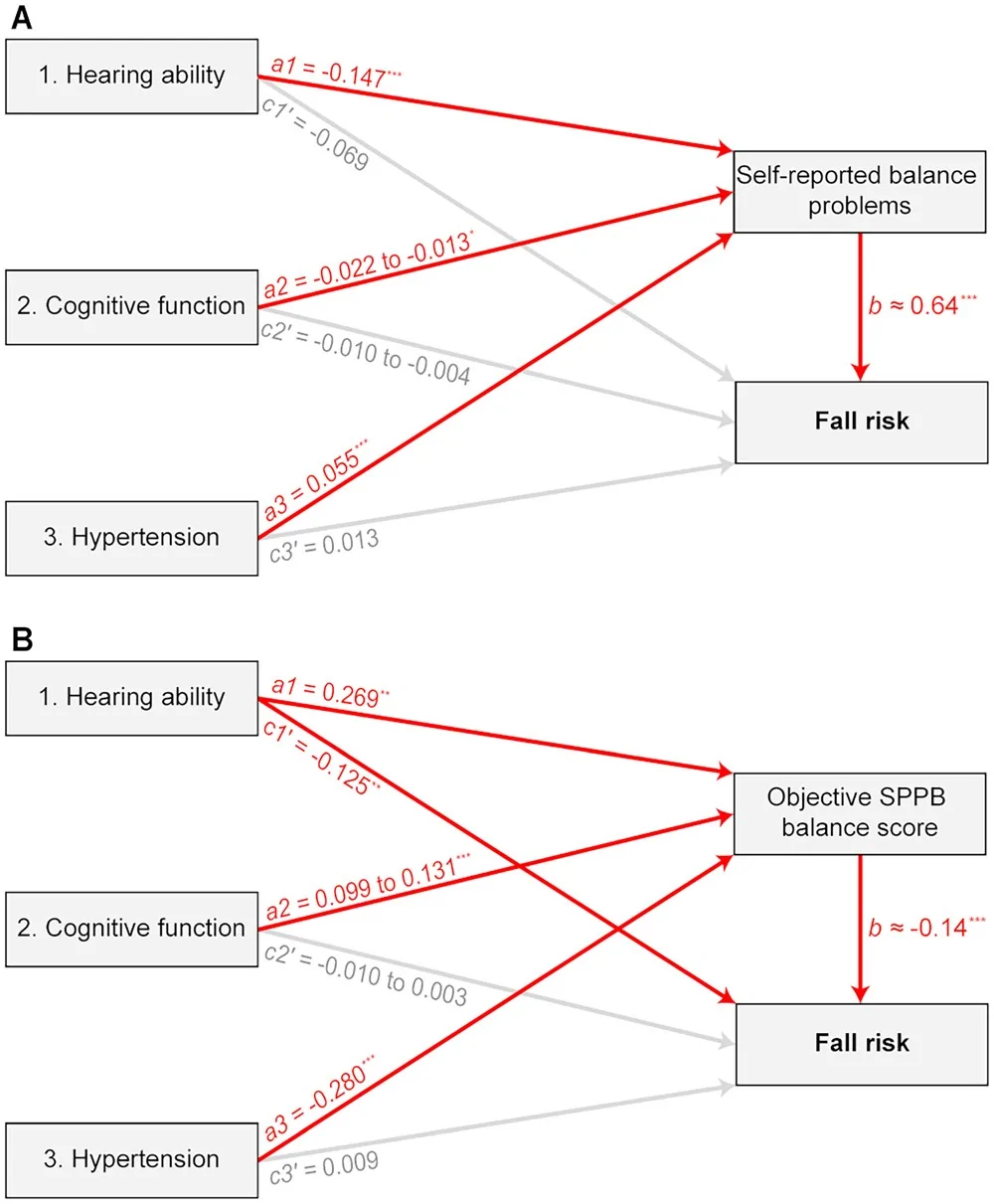
Conceptual diagrams of mediation analyses examining fall risk. Red lines indicate statistically significant relationships (*p < .05; **p < .01; ***p < .001). (A) Self-reported balance problems as the mediator. Full mediation was observed when the predictor was hearing ability, cognitive function, or hypertension. (B) Objective short physical performance battery (SPPB) balance score as the mediator. Full mediation was observed for cognitive function and hypertension, with partial mediation for hearing ability.

When self-reported balance problems were the mediator, full mediation was observed for hearing ability, all cognitive domains, and hypertension (Figure 3A, Supplementary Table 4), with no mediation for heart attack history.

Poorer hearing ability was associated with significantly more balance problems (*a* = –0.147, *p *< .001), which in turn increased fall risk (*b* = 0.639, *p *< .001). After accounting for this pathway, the direct effect of hearing ability on fall risk was no longer significant (*c*′ = –0.069, *p *= .08). The significant indirect effect (*a×b* = –0.094; Sobel *z* = –4.83, *p *< .001) indicates that the relationship between hearing ability and fall risk was fully mediated by self-reported balance.

Similarly, all 4 cognitive domains (executive function, immediate memory, delayed memory, and orientation) demonstrated full mediation. Worse performance in these domains predicted more self-reported balance problems (path a coefficients from −0.022 to −0.013, all *p *< .05), which in turn predicted higher fall risk (*b* ≈ 0.64, *p *< .001). For all 4 domains, the direct effects on fall risk were non-significant (*c*′ from −0.010 to −0.004, all *p *> .05), while the indirect effects were significant (*a×b* ranged from −0.014 to −0.009; Sobel *z* scores ranged from −4.96 to −2.28, all *p *< .05), confirming full mediation.

The presence of hypertension was associated with significantly more self-reported balance problems (*a* = 0.055, *p *< .001), which in turn strongly predicted increasing fall risk (*b* = 0.644, *p *< .001). After accounting for this pathway, the direct effect of hypertension on fall risk was not significant (*c*′ = 0.013, *p *= .60). The significant indirect effect (*a×b* = 0.036, Sobel *z *= 3.47, *p *< .01) confirms that the relationship was fully mediated by self-reported balance.

In contrast, heart attack history did not significantly predict an increase in self-reported balance problems (*a* = 0.039, *p *= .36). While reporting a balance problem remained a strong predictor of increased fall risk (*b* = 0.644, *p *< .001), the lack of a significant association between heart attack history and balance problems resulted in a non-significant indirect effect (*a×b* = 0.025, Sobel *z *= 0.91, *p *= .36). The direct effect of heart attack on fall risk was also not significant (*c*′ = 0.027, *p *= .65).

#### Mediation via objective balance score

When the objective NHATS SPPB balance score was used as the mediator, the findings mirrored those of the subjective balance problems. Full mediation was again observed for all cognitive domains and hypertension, with no mediation for heart attack history. The key distinction was a partial mediation pathway for hearing ability (Figure 3B, Supplementary Table 5).

Better hearing ability significantly predicted better objective balance (*a* = 0.269, *p *< .01), which in turn predicted a lower fall-risk score (*b* = −0.139, *p *< .001). However, a significant direct effect of hearing ability remained (*c*′ = −0.125, *p *< .01) alongside the significant indirect effect (*a×b* = −0.037, Sobel *z *=* −*3.18, *p *< .01), indicating partial mediation.

In contrast, full mediation was consistently observed for all 4 cognitive domains. Higher scores in these domains significantly predicted better objective balance (path a coefficients from 0.099 to 0.131, all *p *< .001), which in turn predicted a lower fall-risk score (*b* ≈ −0.14, *p *< .001). For all 4 domains, the direct effects on fall risk were non-significant (path c′ coefficients from −0.010 to 0.003, all *p *> .05), while the indirect effects were significant (*a×b* from −0.019 to −0.014; Sobel *z* scores from −7.53 to −5.44, all *p *< .001), confirming the full mediation.

The presence of hypertension significantly predicted a lower objective balance score (*a* = −0.280, *p *< .001), which in turn predicted a higher fall-risk score (*b* = −0.142, *p *< .001). A full mediation was confirmed by a non-significant direct effect (*c*′ = 0.009, *p *= .73) and a significant indirect effect (*a×b* = 0.039, Sobel *z *= 5.79, *p *< .001).

Finally, heart attack history showed no evidence of mediation. It did not significantly predict objective balance (*a* = −0.092, *p *= .36), even though better objective balance remained a significant protective factor against falls (*b* = −0.142, *p *< .001). Consequently, the indirect effect on fall risk was not significant (*a×b* = 0.013, Sobel *z *= 0.92, *p *= .36). The direct effect of a prior heart attack was also not significant (*c*′ = 0.039, *p *= .55).

## Discussion

This study used a large, nationally representative sample of older adults to investigate the multifactorial nature of fall risk, shifting the focus from isolated risk factors to a system-level perspective. As hypothesized, upstream domains demonstrated distinct pathways: mental health directly predicted fall risk, whereas hearing and cognitive declines exhibited indirect associations. Our cardiovascular hypothesis was only partially supported, revealing a mechanism-dependent divergence: heart disease showed a direct association with fall risk, but hypertension related indirectly. Crucially, while self-reported balance was the strongest direct predictor, both subjective and objective balance served as convergent mediating pathways linking these sensory, cognitive, and vascular deficits to falls. These results underscore not only the importance of a holistic fall prevention approach addressing both direct risk factors but also the upstream contributors to postural instability.

### The independent role of mental health in fall susceptibility

Depression and anxiety symptoms were found to be significantly associated with a higher fall risk, even after controlling for several physical health conditions and visual ability (Figure 2). This finding aligns with literature suggesting depression is an independent risk factor for falls.^17^^,^^22^^,^^23^ The link between anxiety and falls may be explained by the attentional control theory (ACT),^38^ which posits that anxiety can impair attentional control by shifting focus toward environmental threats instead of task-relevant information.^38^ Consequently, older adults with anxiety may misallocate finite attentional resources during daily activities, compromising the processing required for postural control.^38^ Along with reduced cognitive resources, older adults with anxiety may need to prioritize their daily tasks to prevent falls, for example, stop walking while talking during dual-tasking.^38^ Although we did not explicitly examine the interaction between psychological distress and cognitive function, the results support prior studies indicating that addressing mental health is critical to fall prevention in older adults.^5^^,^^21^^,^^23^^,^^24^^,^^38^

### Balance as a primary predictor and mediator of fall risk

A striking yet anticipated finding was the powerful, independent association of subjective and objective balance measures with fall risk. A self-reported balance problem was the strongest predictor of a higher fall risk (Figure 2), consistent with literature linking self-perceived balance issues to fall history.^39^ Concurrently, better performance on the objective NHATS SPPB balance subtest was significantly protective. This dual finding underscores the role of balance in both functional experience and fall risk in older adults.^15^ Balance appears to serve as a convergent mediating pathway for multiple age-related physiological declines, reinforcing its status as a central clinical target for fall prevention.

#### The complementary value of subjective and objective balance measures

The current study design intentionally included both self-report and objective balance measures, and the results validate this approach. The two measures were moderately correlated (*r *=* −*0.33, *p *< .001; Supplementary Figure 1), confirming that individuals who perceive balance problems perform worse on objective tests.^39^ However, this moderate correlation also indicates that the two measures are not interchangeable. While the objective SPPB score assesses physical capacity under standardized conditions, a self-reported balance problem may reflect physical instability, fear of falling, loss of confidence, and/or activity avoidance, which are powerful risk factors of falls.^22^ That both measures contributed significant and unique variance to the regression model strongly suggests that a more comprehensive picture of fall risk can be obtained by asking patients about their perceived postural stability in addition to conducting performance-based tests.

The observed primacy of subjective perception over objective performance reflects the capacity of self-reported balance measures to capture psychological distress and fear of falling.^23^ When individuals perceive a high risk of falling, fear triggers a reflexive “stiffening strategy,”^25^^,^^26^ and/or drives a “vicious cycle” of activity avoidance.^27^^,^^28^ This fear-driven behavioral cascade consolidates a high-fall-risk phenotype, making subjective perception a highly sensitive predictor of future falls.^28^ It should be noted that an appraisal of a balance problem, which inherently contains elements of “fear of falling,” is not merely a reflection of the “worry about falling” captured by our fall risk score.^23^ While both concepts are rooted in the psychological domain and naturally overlap, literature suggests that fear represents the broader emotional response, whereas worry acts as the specific cognitive component of anxiety that actively drains working memory during movement.^23^^,^^38^ However, because both measures rely on self-perception, they may be concurrently influenced by underlying affective states and previous fall experiences.^23^^,^^28^ Building on this conceptual nuance, our empirical data confirm that self-reported balance problems and the composite fall risk score are not statistically redundant, sharing only ∼20% variance. Thus, perceiving a balance problem offers unique insight into functional fall risk beyond mere worry. Consequently, subjective reports serve as powerful, immediate indicators that often demonstrate high predictive value.^28^

#### Balance as a convergent pathway: mediating the effects of hearing and cognition

A key finding of this study is that, in the fully adjusted model, no measure of hearing or cognitive function was a significant direct predictor of fall risk (Figure 2). Similarly, previous studies using nationally representative U.S. older adult samples found that neither self-reported hearing loss^40^ nor cognitive impairment^17^ significantly increased fall risk after adjusting for confounders. The present findings echo these studies and support the significance of their notion about including important fall risk confounders. However, the mediation analysis revealed hearing and cognition as upstream contributors to balance problems, positioning balance as the convergent mediating mechanism through which these factors relate to fall risk (Figure 3). The indirect association of hearing and cognition with fall risk was almost entirely accounted for by balance. This link is not surprising, given the relationship between hearing impairment and SPPB balance performance in community-dwelling older adults.^14^ Moreover, in a longitudinal study using the 2011-2018 NHATS data, cognitive impairment measured using the CDT and immediate/delayed recall predicted the reduction of the NHATS SPPB score, which is strongly associated with fall risk.^17^ The present findings explain the importance of hearing and cognitive integrity: they act as foundational pillars for maintaining postural control. When these systems decline, a critical manifestation is a balance problem, which is subsequently associated with increased fall risk.^12^^,^^41^

For all cognitive domains, a pattern of full mediation was observed. Better cognitive scores across executive function, memory, and orientation were associated with better balance (both subjective and objective), which in turn predicted lower fall risk. Notably, the direct association between cognitive domains and fall risk was rendered non-significant, which indicates the relationship between cognitive domains and fall risk is fully mediated by balance (Figure 3). This supports cognitive-motor integration, where a diverse central neural processing is essential for planning and executing stable movement.^15^ As older adults tend to prioritize motor control over other tasks to prevent falls during essential daily activities, greater preservation of cognitive orientation, executive function, and memory performance may reduce cognitive-motor interference and thereby limit the risk of postural sway.^18^^,^^42^

The findings of indirect associations for the memory domains are particularly clarifying, given their previously inconsistent links to fall risk.^16^^,^^18^^,^^19^ Immediate memory serves as a direct input of the real-time environmental encoding for the postural control system.^18^^,^^20^ When this moment-to-moment cognitive encoding falters, balance problems can result, increasing fall risk. Similarly, delayed memory serves a protective role, likely through the recall of past spatial experiences to navigate anticipated balance challenges.^19^ Therefore, the present model reframes these memory domains, showing that their relationship with safe ambulation operates through the mechanism of balance.

Interestingly, hearing ability demonstrated partial mediation when objective balance was the mediator (Figure 3B). While a significant portion of hearing’s protective effect was channeled through better objective balance, a smaller, direct protective effect remained. This suggests that good hearing may contribute to fall prevention beyond those captured by the SPPB balance test alone, perhaps by enhancing spatial awareness and reactions to auditory cues in the environment.^43^ In contrast, self-reported balance problems served as a full mediator, likely because they reflect a comprehensive picture of stability, including the environmental awareness provided by hearing.^12^^,^^44^

### The nuanced relationship between selected cardiovascular conditions and fall risk

The present results suggest a complex relationship between selected cardiovascular conditions and falls. While a history of heart disease directly predicted higher fall risk (Figure 2), hypertension and a history of heart attack did not. However, mediation analysis revealed that hypertension significantly increased fall risk indirectly by worsening both subjective and objective balance (Figure 3A and B). This suggests the impact of vascular risk factors on falls is not uniform, as noted in previous reviews.^31^^,^^32^^,^^45^ Chronic conditions like heart disease may contribute to frailty that directly increases fall risk, whereas the impact of hypertension may be more insidious, gradually damaging the vestibular, cerebral, and peripheral nervous systems that are essential for maintaining balance.^9^ While the role of antihypertensive medications in fall risk is still disputed,^46^ hypertension itself is linked to cerebral microvascular damage that can degrade white matter tracts essential for cognitive and motor control, thereby impairing balance and indirectly precipitating falls.^9^^,^^46^ This underscores the need to manage vascular health not only for cardiovascular outcomes but also for fall prevention.

### Clinical implications

The present findings suggest a comprehensive approach to assessing fall risk, particularly for older adults with hearing loss. Given that patients with hearing and vestibular disorders fall more frequently than their peers,^5^^,^^13^ healthcare providers should implement routine fall risk screening for this population. A thorough assessment in older adults should extend beyond balance by incorporating hearing, cognitive, mental health, and vascular status. These findings corroborate recent guidelines from the World Falls Guidelines Task Force,^47^^,^^48^ which recommend a multifactorial falls risk evaluation that incorporates assessments of gait, balance, cognition, fall concerns, cardiovascular disorders, vestibular disorders, sensory deficits, anxiety, and depression for older adults at high fall risk. This approach is critical for conditions that involve both hearing loss and vestibular dysfunction,^49^ such as Meniere’s disease, to ensure contributing factors of fall risk are identified for patient-centered intervention.

### Limitations

This study has several limitations. First, the cross-sectional design precludes establishing causal relationships. Second, by including only community-dwelling older adults, the findings cannot be generalized to individuals in nursing homes or other residential facilities. Third, excluding individuals with severe neurological or sensory pathologies (e.g., dementia, stroke, and deafness) reduced confounding but limits generalizability to relatively higher-functioning community-dwelling older adults. Notably, the indirect associations observed for hearing and cognition may reflect a restriction of range; populations with more profound impairments might exhibit direct associations with fall risk. Fourth, listwise deletion introduced modest selection bias (Supplementary Table 1). Consequently, findings may be subject to nonresponse bias and primarily represent this analytic subpopulation rather than the entire NHATS cohort. Fifth, the reliance on self-reported data potentially introduces recall and response biases and may influence the observed associations. For example, hearing ability was based on self-report rather than measured hearing thresholds. While self-reported hearing difficulty is clinically relevant to fall risk,^50^ it may not perfectly align with clinical measures and thus may underestimate the association between hearing loss and functional outcomes.^44^ Although not attempted by all respondents, NHATS incorporated pure tone audiometry since Round 11 to measure hearing acuity; future endeavors using these data will be invaluable for understanding how hearing thresholds impact falls. Sixth, without a comprehensive medication list, the effect of medications or polypharmacy on the chronic conditions we examined cannot be ruled out.^23^ For instance, while an indirect association of hypertension with fall risk was identified, we lacked information on specific antihypertensive medications or their dosages, which may act as confounders.^46^ Lastly, the current analysis focused on several variables relevant to falls, with adjustments for covariates. While this concentrated analysis facilitates interpretation, it also hinders the examination of other relevant variables of falls, such as smoking, pain, living environment, and nutritional status.^48^ Crucially, we did not model musculoskeletal or mobility-related contributors (e.g., muscle weakness, gait biomechanics) as primary predictors. Although adjusted for, arthritis and osteoporosis only partially proxy these structural domains. Future studies should integrate these mechanical factors to capture a comprehensive fall model.

## Conclusion and future directions

In conclusion, this study identifies a fall risk model highlighting the distinct roles of modifiable psychological and physical factors for fall prevention or intervention. The present findings demonstrate that while mental health problems are directly associated with fall risk, the indirect associations of hearing impairment, cognitive decline, and certain cardiovascular conditions with fall risk are consistent with a model where balance serves as a convergent mediating pathway for upstream physiological deficits. This suggests that future research should adopt an integrated, systems-level approach to advance fall prevention, investigating the broad multisystem interplay governing postural stability and the central mediating role of balance. Such work, particularly incorporating precise clinical measures like audiometry, cognitive assessments, and hemodynamic monitoring, is an important step toward developing effective, multimodal interventions that can accurately predict and ultimately aim to reduce the burden of falls in the aging population.

## Supplementary Material

igag076_Supplementary_Data

## Data Availability

This study analyzed publicly available data from the National Health and Aging Trends Study (NHATS). These data can be accessed directly at https://www.nhats.org. The computer code used for the statistical analysis is available from the corresponding author (Dr Somayeh Shahsavarani) upon reasonable request.
