# Supplementary material for: Balance bridges upstream deficits associated with falls in older adults: evidence from the National Health and Aging Trends Study Round 13 cohort
Source: Innov Aging. 2026 Jul 1;10(10):igag076. doi: 10.1093/geroni/igag076 (PMC13570676; doi:10.1093/geroni/igag076)
Supplement: Supplementary file 1 [file igag076_supplementary_data.pdf]

***Innovation in Aging* Supplementary Material: Shahsavarani & Tai. Balance bridges upstream deficits associated with falls in older adults: Evidence from the National Health and Aging Trends Study Round 13 cohort.**

Supplementary Methods

Survey-Weighted Correlation Analysis

To calculate correlation between self-reported balance and objective balance performance while accounting for unequal sampling probabilities, we estimated a survey-weighted Pearson correlation coefficient. Let x_i denote the self-reported balance score, y_i the objective balance score, and w_i the analytic weight for participant i. The survey-weighted Pearson correlation was computed using weighted means, covariance, and variances[1]:

$$\bar{x}_w = \frac{\sum w_i x_i}{\sum w_i},$$

$$\bar{y}_w = \frac{\sum w_i y_i}{\sum w_i},$$

$$Cov_w(x, y) = \frac{\sum w_i (x_i - \bar{x}_w)(y_i - \bar{y}_w)}{\sum w_i},$$

$$Var_w(x) = \frac{\sum w_i (x_i - \bar{x}_w)^2}{\sum w_i},$$

$$Var_w(y) = \frac{\sum w_i (y_i - \bar{y}_w)^2}{\sum w_i}.$$

The weighted correlation was then defined as:

$$r_w = \frac{Cov_w(x, y)}{\sqrt{Var_w(x) Var_w(y)}}.$$

Because unequal weighting reduces the effective information content of the sample, statistical inference was based on a weight-adjusted effective sample size[2,3]:

$$n_{eff} = \frac{(\sum w_i)^2}{\sum w_i^2}.$$

The quantity n_{eff} approximates the effective degrees of freedom available for inference after accounting for unequal weighting and corresponds to the classical Kish effective sample size. A large-sample *t* approximation was then used to test the null hypothesis H_0 $(r_w = 0)$:

$$t = r_w \sqrt{\frac{n_{eff} - 2}{1 - r_w^2}},$$

with degrees of freedom $df = n_{eff} - 2$. Two-tailed p-values were obtained from the *t* distribution.

Sensitivity Analysis for the Choice of Linear Regression

The primary outcome of this study, the composite fall-risk score, is a bounded 4-point ordinal scale (ranging from 0 to 3). Mathematically, ordinal models such as ordered logistic regression are traditionally designed for this type of discrete, ranked data. However, we intentionally selected survey-weighted linear regression as our primary analytical approach. This decision was made because linear regression produces coefficients that are highly intuitive and directly interpretable for a general clinical audience. Furthermore, established statistical literature demonstrates that when an ordinal scale has four or more categories and the sample size is large (our sample is N = 4,805), linear regression is highly robust and rarely alters substantive conclusions.[4,5] To explicitly prove that our choice of linear regression was appropriate, we conducted a sensitivity analysis by re-running our primary model using an ordered logistic regression.

The ordered logistic regression yielded results that perfectly mirrored the linear regression model in both directionality and statistical significance. The predictors that were significant in the main text remained highly significant in the ordinal model: self-reported balance problems (Proportional Odds Ratio [OR] = 4.84, 95% CI: 4.19–5.58), objective balance scores (Proportional OR = 0.78, 95% CI: 0.74–0.83), depression (Proportional OR = 1.17, 95% CI: 1.10–1.24), anxiety (Proportional OR = 1.20, 95% CI: 1.14–1.28), and heart disease (Proportional OR = 1.28, 95% CI: 1.09–1.50). Just as importantly, variables that were non-significant in our primary linear model, such as hearing ability, individual cognitive domains, history of heart attack, and hypertension, remained non-significant in the ordinal model.

The alignment of findings between the ordered logistic and linear regression models indicates that the overarching conclusions are robust to model specification. Given that the use of a linear model for the discrete outcome did not alter the significance or directionality of the primary predictors, the survey-weighted linear regression was maintained in the primary analysis to provide clinically interpretable coefficients.

Sensitivity Analysis of the Composite Fall-Risk Score

We operationalized the primary outcome as a composite score to assess cumulative fall risk. This approach aligns with established clinical guidelines, such as the Centers for Disease Control and Prevention's (CDC) Stopping Elderly Accidents, Deaths, and Injuries (STEADI) algorithm, which incorporates both fall history and worry about falling as primary screening criteria for risk stratification.[6] An ordinal score ranging from 0 to 3 provides a measure of cumulative risk severity, offering greater statistical variability than isolated binary items. Methodologically, these specific items were selected to minimize the structural missing data introduced by the skip-logic of follow-up questions, such as those regarding recurrent falls or

activity limitations. Finally, prior literature demonstrates that physical falls and the fear of falling are highly correlated and bidirectionally associated, supporting their integration into a single composite measure of overall fall risk.[7,8]

To verify the robustness of our primary outcome measure, the ordinal composite fall-risk score, we conducted sensitivity analyses evaluating its three individual components independently: experiencing a fall in the past month, experiencing a fall in the past year, and worrying about falling. Because these isolated outcomes are binary rather than ordinal, survey-weighted logistic regression models (Generalized Linear Models with a binomial family and logit link function) were utilized in place of weighted least squares. All models incorporated the NHATS survey weights and heteroskedasticity-consistent robust standard errors to maintain national representativeness and account for the complex survey design. To ensure accurate variance estimation within the generalized linear modeling framework, these survey weights were normalized to the analytic sample size prior to model fitting. To facilitate clinical interpretation, the resulting log-odds coefficients were exponentiated and reported as Odds Ratios (ORs) alongside their 95% Confidence Intervals (CIs). These sensitivity models adjusted for the exact same covariates and upstream domains as the primary multivariable analysis, with results detailed in Supplementary Table 3.

Mediation Analysis

Predictors not significantly associated with the fall-risk score in the full regression model were subsequently examined for mediation by balance. Two parallel mediation models using either self-reported balance problems or the objective NHATS SPPB balance score as the mediator were conducted. All mediation analyses were carried out using WLS with HC3 robust standard errors. Each mediation model estimated three key pathways:

1. **Path a: Predictor → Mediator.** The association between the predictor of interest and the mediator (balance) was estimated, controlling for all covariates. This path quantified the predictor's influence on balance.
2. **Path b: Mediator → Fall Risk.** The association between the mediator and the fall-risk score was estimated, controlling for the predictor and all covariates.
3. **Path c′: Predictor → Fall Risk, controlling for Mediator.** The direct effect of the predictor on the fall-risk score was estimated after accounting for the mediator and covariates. This path represents the direct effect of a predictor on fall risk.

The product of coefficients from paths a and b represents the indirect effect (Predictor → Balance → Fall Risk). This pathway was tested using the Sobel test, which evaluates if the product of the two regression coefficients is significantly different from zero and combines the standard errors of both paths to determine if the indirect effect is statistically significant. Results were interpreted as follows:

- **Full mediation:** A significant indirect effect (significant paths a and b), and a non-significant direct effect (path c′), indicating that the predictor influenced fall risk entirely through balance.
- **Partial mediation:** Significant paths a, b, and c′, indicating the predictor affected fall risk both directly and indirectly.
- **No mediation:** Either path a or the indirect effect was not significant.

Rationale for the Sobel Test in Survey-Weighted Mediation Analysis

While landmark research[9] notes that the product of coefficients ($\alpha\beta$, representing the $a \times b$ indirect effect in the current study) is fundamentally non-normal, the Sobel test was selected for two primary reasons: design-based consistency and large sample robustness.

Design-Based Consistency. Because the NHATS utilizes a complex sample design with analytic weights and stratification, a design-based estimation framework was essential to ensure results are representative of the U.S. older adult population. The Sobel test utilizes the multivariate delta method, which serves as the theoretical basis for Taylor Series Linearization (TSL). As noted by Heeringa et al,[3] TSL is a foundational approach for design-based estimation that integrates complex analytic weights and robust standard errors directly into the variance estimation process. While contemporary replication methods such as the bootstrap are also valid for complex surveys,[3] the TSL/Delta method approach was prioritized to maintain the integrity of the NHATS survey-weighting structure within a stable, asymptotic framework, avoiding the technical complexity of specialized resampling required to preserve the survey structure.

Large Sample Robustness. In the current large analytic sample (n = 4,805), the Sobel test's reliance on the normal distribution provides a conservative estimate of significance. MacKinnon et al[9] noted that while the normality assumption for the product of coefficients is technically inappropriate due to the asymmetric and high-kurtosis nature of the distribution, the resulting conservative critical values based on the normal sampling distribution often "compensate for the inflation in the Type I error rate" associated with the compound form of the null hypothesis. Furthermore, because the relative bias in point estimates of the indirect effect was shown to decrease as sample size increased in simulations up to n = 1,000,[9] and the current sample size is more than four times larger than that maximum, the Sobel test was deemed the most reliable and statistically cautious method for this specific complex-survey context.

Supplementary Tables

Supplementary Table 1. Comparison of Demographic and Health Characteristics Between the Analytic Sample and Participants Excluded Due to Missing Data

Characteristics	Included % (n = 4,805)	Excluded % (n = 1,092)	Δ%[a]	*p*-value[b]
**Gender**				0.002
Male	42.7	37.5	-5.2	
Female	57.3	62.5	5.2	
**Age (years)**				< 0.001
65-69	13.5	13.6	0.1	
70-74	24.4	18.3	-6.1	
75-79	25.1	22.7	-2.4	
80-84	20.2	21.6	1.4	
85-89	10.5	13.0	2.5	
≥90	6.2	10.8	4.6	
**Education**				< 0.001
No schooling completed	1.0	2.6	1.6	
1st-8th grade	7.6	14.2	6.6	
9th-12th grade (no diploma)	8.4	11.5	3.1	
High school graduate	22.7	23.4	0.7	
Vocational diploma	7.4	6.5	-0.9	
Some college but no degree	14.8	13.1	-1.7	
Associate's degree	7.1	5.4	-1.7	
Bachelor's degree	15.6	11.6	-4.0	
Master's or above	15.4	11.7	-3.7	
**History of chronic conditions**				
Arthritis (yes)	61.9	71.5	9.6	< 0.001
Osteoporosis (yes)	27.1	32.0	4.9	0.001
Lung disease (yes)	18.6	22.3	3.7	0.007
Heart attack (yes)	3.6	6.5	2.9	< 0.001
Heart disease (yes)	18.0	21.8	3.8	0.004
Hypertension (yes)	68.9	73.3	4.4	0.004

[a] Percentage difference (Excluded % – Included %).
[b] For Gender and Chronic Conditions, *p*-values were derived from Pearson's Chi-square tests comparing the proportion of "Yes" responses between groups. For Age Range and Education Level, a single Global Chi-square test of independence was performed to evaluate differences across the entire frequency distribution of categories

Supplementary Table 2. Results of Variance Inflation Factor (VIF) Analysis[a]

Predictors	VIF
Hearing ability	1.08
Self-reported balance problem	1.26
Objective SPPB balance score[b]	1.43
Executive function	1.26
Immediate memory	2.32
Delayed memory	2.30
Orientation	1.39
Depression	1.57
Anxiety	1.52
Heart attack history	1.05
Heart disease history	1.10
Hypertension	1.06
Age range	1.26
Gender	1.25
Education level	1.32
Visual ability	1.08
Arthritis	1.16
Osteoporosis	1.24
Lung disease	1.07

[a]See Reference Kim[10] for methodological details.
[b]NHATS Expanded Short Physical Performance Battery (SPPB)

Supplementary Table 3. Sensitivity Analysis of Individual Fall-Risk Components using Weighted Logistic Regressions

Predictor	Falling in Last Month: OR (95% CI)[a]	Falling in Last Year: OR (95% CI)	Worry about Falling: OR (95% CI)
Hearing ability	1.09 (0.82 – 1.44)	1.10 (0.88 – 1.39)	0.81 (0.65 – 1.01)
Self-reported balance problem	3.65 (2.93 – 4.55)	2.73 (2.32 – 3.22)	5.27 (4.53 – 6.13)
Objective SPPB balance score[b]	0.81 (0.73 – 0.89)	0.87 (0.81 – 0.94)	0.70 (0.65 – 0.75)
Executive function	1.01 (0.91 – 1.13)	1.06 (0.98 – 1.14)	0.98 (0.91 – 1.06)
Immediate memory	0.90 (0.82 – 0.99)	1.11 (1.04 – 1.18)	0.99 (0.92 – 1.05)
Delayed memory	1.14 (1.06 – 1.23)	0.96 (0.91 – 1.02)	1.00 (0.94 – 1.05)
Orientation	0.90 (0.83 – 0.97)	0.98 (0.92 – 1.04)	1.09 (1.03 – 1.16)
Depression	1.08 (1.00 – 1.17)	1.08 (1.01 – 1.16)	1.19 (1.11 – 1.27)
Anxiety	1.10 (1.01 – 1.20)	1.10 (1.02 – 1.18)	1.14 (1.07 – 1.23)
Heart attacke	1.36 (0.91 – 2.03)	0.82 (0.57 – 1.19)	1.09 (0.78 – 1.53)
Heart disease	0.83 (0.64 – 1.09)	1.24 (1.03 – 1.50)	1.11 (0.91 – 1.34)
Hypertension	0.76 (0.62 – 0.93)	0.91 (0.78 – 1.05)	1.07 (0.92 – 1.25)

[a]OR = Odds Ratio; CI = Confidence Interval
[b]NHATS Expanded Short Physical Performance Battery (SPPB)

Supplementary Table 4. Summary of the Mediation Analysis Results Using Self-Reported Balance Problem as the Mediator

**Predictor**	**Effect of Predictor on Mediator (*a*)**	**Effect of Mediator on Fall Risk (*b*)**	**Indirect Effect (*a x b*)**	**Direct Effect (c')**
Hearing ability	-0.147***	0.639***	-0.094***	-0.069
Executive function	-0.018*	0.644***	-0.012*	-0.009
Immediate memory	-0.018**	0.644***	-0.011**	-0.004
Delayed memory	-0.022***	0.641***	-0.014***	-0.010
Orientation	-0.013*	0.644***	-0.009*	-0.004
Hypertension	0.055***	0.644***	0.036**	0.013
Heart attack	0.039	0.644***	0.025	0.027

*$p < 0.05$; **$p < 0.01$; ***$p < 0.001$

Supplementary Table 5. Summary of the Mediation Analysis Results Using Objective Balance Score as the Mediator

**Predictor**	**Effect of Predictor on Mediator (*a*)**	**Effect of Mediator on Fall Risk (*b*)**	**Indirect Effect (*a x b*)**	**Direct Effect (c')**
Hearing ability	0.269**	-0.139***	-0.037**	-0.125**
Executive function	0.124***	-0.142***	-0.018***	-0.003
Immediate memory	0.131***	-0.143***	-0.019***	0.003
Delayed memory	0.099***	-0.139***	-0.014***	-0.010
Orientation	0.105***	-0.142***	-0.015***	0.003
Hypertension	-0.280***	-0.142***	0.039***	0.009
Heart attack	-0.092	-0.142***	0.013	0.039

*$p < 0.05$; **$p < 0.01$; ***$p < 0.001$

Supplementary Figures

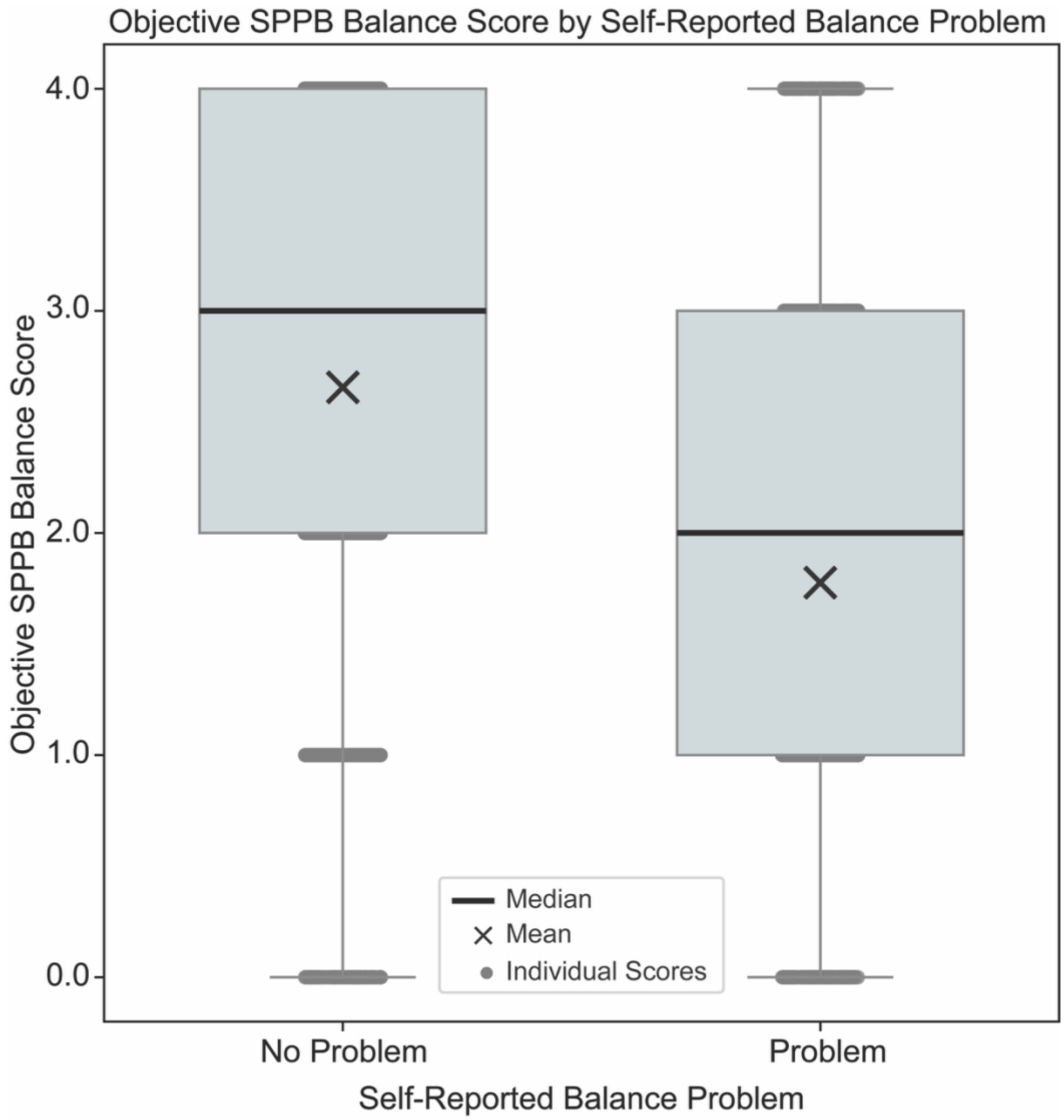

Supplementary Figure 1. Boxplot showing the relationship between objective balance score and self-report balance problem. A moderate inverse relationship was found between objective balance score and self-report balance problem (r = -0.331, $p < 0.001$). The objective measure was the score from the balance subset of the NHATS Expanded Short Physical Performance Battery (SPPB).

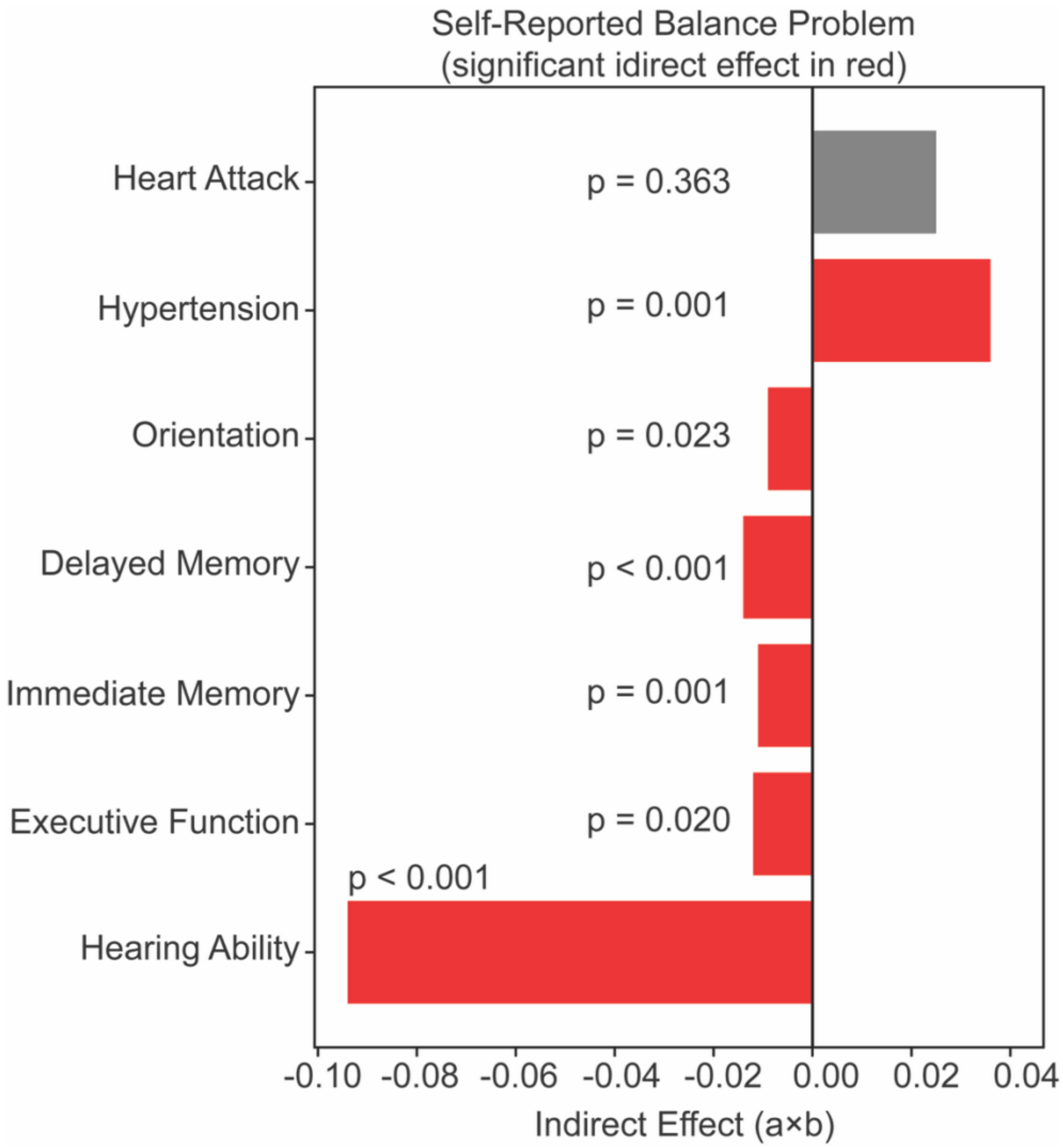

Supplementary Figure 2. Results of the mediation analyses showing the indirect effects of predictors on the fall risk score, with self-reported balance problems being the mediator. Each bar represents the magnitude of the indirect effect (a × b), with red bars indicating a statistically significant pathway ($p < 0.05$).

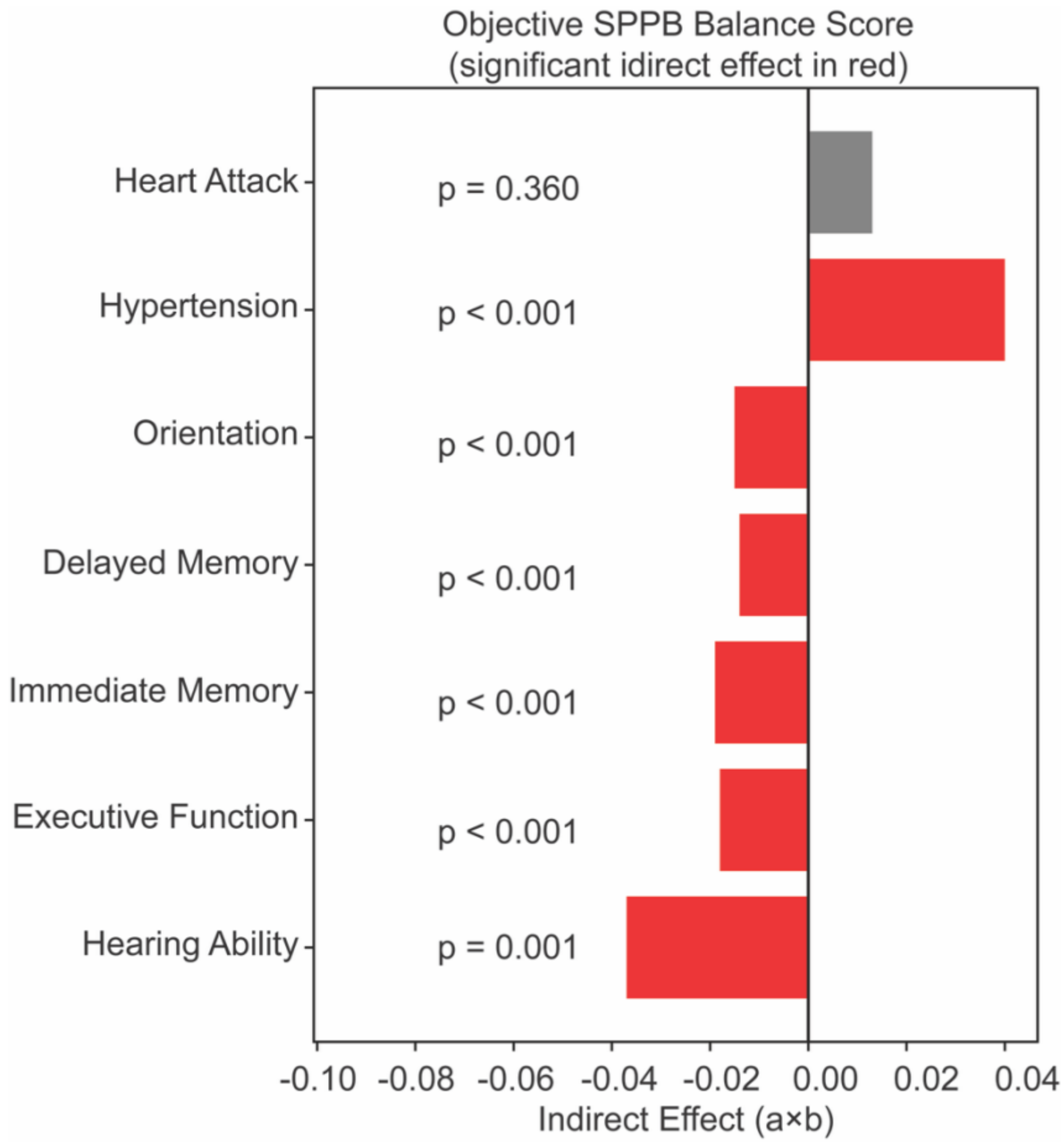

Supplementary Figure 3. Results of the mediation analyses showing the indirect effects of predictors on the fall risk score, with objective balance score being the mediator. Each bar represents the magnitude of the indirect effect (a × b), with red bars indicating a statistically significant pathway ($p < 0.05$). The objective measure was the score from the balance subset of the NHATS Expanded Short Physical Performance Battery (SPPB).

References

1. Bailey P, Emad A, Zhang T, Xie Q, Sikali E. Weighted and Unweighted Correlation Methods for Large-Scale Educational Assessment: wCorr Formulas. AIR–NAEP Working Paper No. 2018-01. NCES Data R Project Series# 02. *American Institutes for Research*. Published online 2018. Accessed March 4, 2026. https://eric.ed.gov/?id=ED585538

2. Kish L. Survey sampling. Published online 1965. Accessed March 4, 2026. https://psycnet.apa.org/record/1965-35018-000?utm_medium=email&utm_source=transaction

3. Heeringa SG, West BT, Heeringa SG, Berglund PA. *Applied Survey Data Analysis*. chapman and hall/CRC; 2017. Accessed March 4, 2026. https://www.taylorfrancis.com/books/mono/10.1201/9781315153278/applied-survey-data-analysis-steven-heeringa-patricia-berglund-brady-west-steve-heeringa-patricia-berglund

4. Norman G. Likert scales, levels of measurement and the “laws” of statistics. *Advances in health sciences education*. 2010;15(5):625-632.

5. Rhemtulla M, Brosseau-Liard PÉ, Savalei V. When can categorical variables be treated as continuous? A comparison of robust continuous and categorical SEM estimation methods under suboptimal conditions. *Psychological methods*. 2012;17(3):354.

6. Crow RS, Lohman MC, Pidgeon D, Bruce ML, Bartels SJ, Batsis JA. Frailty Versus Stopping Elderly Accidents, Deaths and Injuries Initiative Fall Risk Score: Ability to Predict Future Falls. *J American Geriatrics Society*. 2018;66(3):577-583. doi:10.1111/jgs.15275

7. Chen WC, Li YT, Tung TH, Chen C, Tsai CY. The relationship between falling and fear of falling among community-dwelling elderly. *Medicine*. 2021;100(26):e26492.

8. Ehrlich JR, Hassan SE, Stagg BC. Prevalence of Falls and Fall-Related Outcomes in Older Adults with Self-Reported Vision Impairment. *J American Geriatrics Society*. 2019;67(2):239-245. doi:10.1111/jgs.15628

9. MacKinnon DP, Lockwood CM, Hoffman JM, West SG, Sheets V. A comparison of methods to test mediation and other intervening variable effects. *Psychological methods*. 2002;7(1):83.

10. Kim JH. Multicollinearity and misleading statistical results. *Korean journal of anesthesiology*. 2019;72(6):558-569.
